# Catatonia, beyond a psychiatric syndrome

**DOI:** 10.1590/1980-57642016dn11-020015

**Published:** 2017

**Authors:** Nathália Stela Visoná de Figueiredo, Débora Bartzen Moraes Angst, Antônio de Matos Lima Neto, Michel Ferreira Machado, Maria Sheila Guimarães Rocha, Sônia Maria Dozzi Brucki

**Affiliations:** 1 MD, Neurologist, Hospital Santa Marcelina, Department of Neurology.; 2 MsC Neurologist, Hospital Santa Marcelina, Department of Neurology.; 3 PhD Neurologist, Hospital Santa Marcelina, Department of Neurology.

**Keywords:** catatonia, intracranial sinus thrombosis, brain infarction, status epilepticus, catatonia, trombose dos seios intracranianos, infarto encefálico, estado epiléptico

## Abstract

Although catatonia is a well-known psychiatric syndrome, there are many possible systemic and neurological etiologies. The aim of this case report was to present a case of a patient with cerebral venous sinus thrombosis and infarction in which catatonia was the clinical manifestation of a possible nonconvulsive status epilepticus. To our knowledge, only one such case has been reported in the literature, which had a simplified diagnostic investigation. It is important to correctly recognize the organic cause underlying catatonia in order to treat the patient as soon as possible thereby improving outcome. Therefore, physicians need to update their knowledge on catatonia, recognizing that it can be part of a psychiatric or neurologic condition.

## Abstract (pt)

Embora a catatonia seja uma síndrome psiquiátrica bem conhecida, existem várias etiologias possíveis, tanto sistêmicas quanto neurológicas. O objetivo deste relato de caso é apresentar um quadro de trombose venosa central com infarto venoso em que a catatonia foi a manifestação clínica de um possível *status* não convulsivo. Na concepção dos autores, apenas um caso é encontrado na literatura, porém com uma propedêutica simplificada. É importante o correto reconhecimento das causas orgânicas que podem estar causando a catatonia para que sejam corrigidas assim que possível, melhorando o prognóstico do paciente. Além disso, os médicos precisam atualizar seus conhecimentos sobre a catatonia, uma vez que ela pode ser parte tanto de uma condição psiquiátrica quanto neurológica.

## INTRODUCTION

Historically, catatonia is associated with schizophrenia, a psychiatric disorder. However, many different etiologies can be implicated, ranging from neurologic to systemic diseases.^1^ In fact, there are many different criteria for defining catatonia as a syndrome, and divergence exists among specialists as to the most appropriate definition for describing it as a separate entity. However, all definitions share some aspects. Thus, a diagnosis of catatonia requires at least one motor component associated with behavioral symptoms.^2-4^ We report a rare case of a patient with catatonia as the initial clinical picture of cerebral venous sinus thrombosis (CVST) in association with cerebral infarction. Moreover, in the present case, catatonia was likely part of a nonconvulsive status epilepticus (NCSE) secondary to CVST. To our knowledge, only one similar report has been published in literature to date, although causal factors and clinical evolution in the present case are reported in more detail.

## CASE REPORT

A 45-year-old man presented at the Emergency Department with a 3-day history of an intense frontal headache with nausea plus temporal and spatial disorientation. The symptoms progressively worsened and were accompanied by inappropriate behavior.

At admission, the patient was afebrile with mild hypertension (170/100 mmHg). On initial neurological examination, he had spontaneous eye opening but reduced blink rate. He exhibited lack of spontaneous movements, mutism, as well as waxy flexibility, negativism and catalepsy, clinical features consistent with catatonia as a syndrome. However, there was no motor impairment such as cranial nerve paralysis. Additionally, deep tendon reflexes were normal and pupils symmetric and reactive to light. He had repetitive stereotypic perioral movements and clonic jerks in the right upper limb lasting for more than five minutes, interpreted as a continuous partial motor status with impairment of consciousness.

After prompt treatment with intravenous Phenytoin (20 mg/kg), he showed some improvement in interaction and total remission of abnormal movements. He had no further clinical signs of catatonia from this point forth throughout follow up. Moreover, he was able to obey verbal commands such as opening and closing his eyes or moving his hand on command. However, he could not produce any verbal words spontaneously, but could understand the physician's instructions. We classified these symptoms as a motor aphasia. Because the patient was examined in an Emergency Room, a more in-depth cognitive evaluation, using validated exams for example, could not be performed.

**Figure 1:**
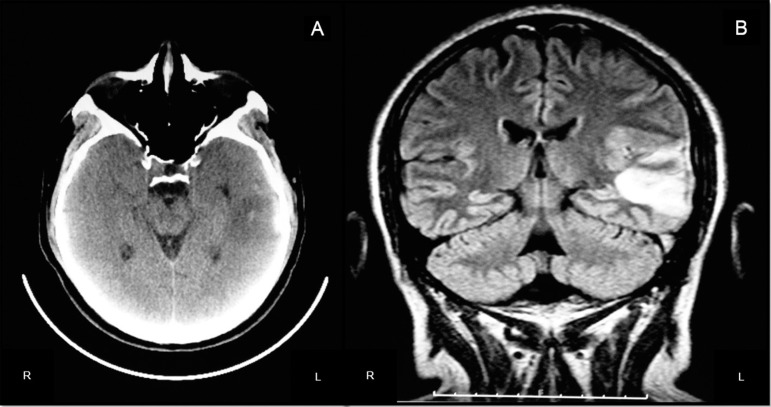
[A] Brain CT from admission showing small hyperdense spots within hypodense area in left temporal lobe. [B] At second day, Flair MRI sequence showed hyperintense image in left posterior temporal lobe, suggesting a venous temporal infarct.

The laboratory exams conducted at the Emergency Department revealed no signs of infection or metabolic disturbance. However, brain computed tomography (CT) disclosed a hypodense image in the left temporal region with small hyperdense spots (Figure 1).

**Figure 2:**
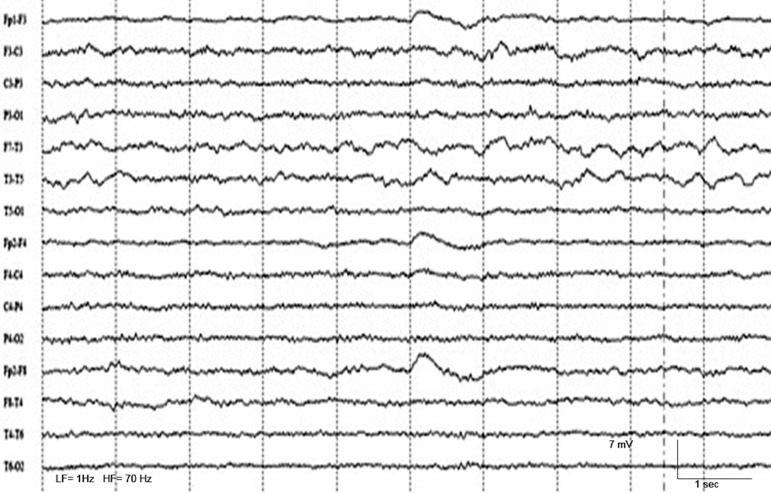
EEG performed on the second day at emergency department revealing diffuse disorganization of the cerebral activity with slow waves on left medium-posterior temporal region, involving electrodes T3 and T5, without epileptic activity. Clinical manifestations had totally disappeared after Phenytoin administration, which was before this EEG recording.

The cerebral spinal fluid (CSF) examination showed six cells/mL, glucose level of 56 and total protein of 27 mg/dL, while cultures for bacteria and fungus were negative. On the second day of hospitalization, electroencephalogram (EEG) showed diffuse disorganization of cerebral activity with predominantly slow waves in delta frequency in the left-posterior medial temporal region and no epileptic activity was recorded (Figure 2).

**Figure 3:**
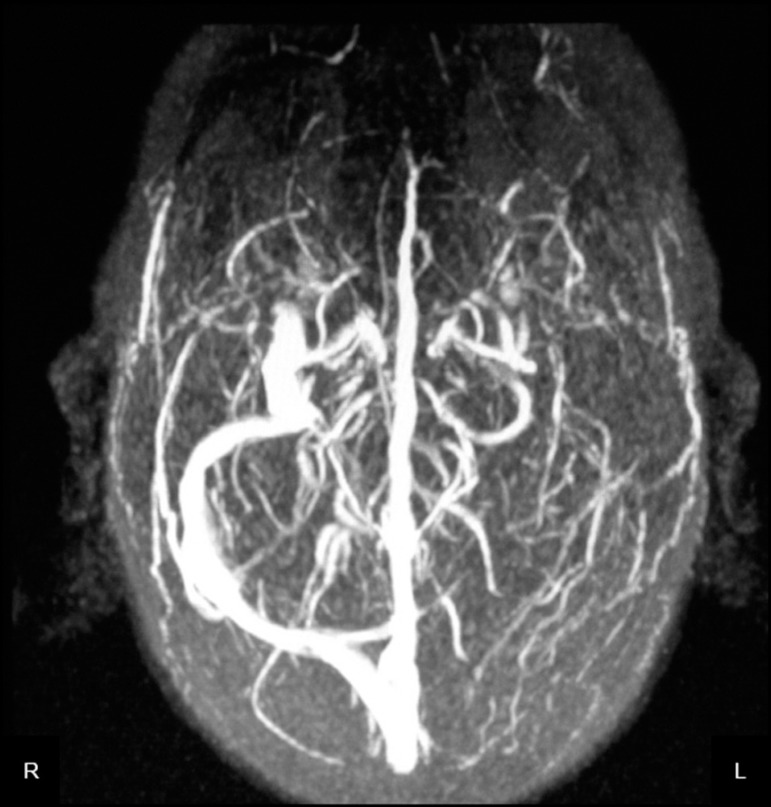
Venous brain MRI performed at second day with filling failure from left transverse sinus to ipsilateral jugular vein due to a cerebral venous sinus thrombosis.

Venous brain magnetic resonance imaging (MRI), also performed on the second day, demonstrated a hyperintense image on T2-weighted and Flair sequences in the left posterior temporal lobe with restriction in DWI sequence without contrast enhancement. Moreover, an absence of flow in the left transverse sinus was evident, suggesting a CVST with adjacent venous temporal infarction (Figure 3). Despite starting anticoagulation with Enoxaparin (1mg/kg/twice a day), motor aphasia persisted. In the short follow-up, no seizures occurred.

## DISCUSSION

According to Barnes et al. (1986), the definition of acute catatonic syndrome (ACS) requires that, over a period of days or weeks, the patient develops at least one motor sign (catalepsy, posturing or waxy flexibility) in combination with one or more behavioral signs such as: (a) negativism, mutism or stupor; (b) excitement; (c) bizarre repetition behavior.^2^ More recent revised criteria from the Diagnostic and Statistical Manual of Mental Disorders 5 (DSM-5) published in 2013 also include agitation, mannerisms, stereotypies, grimacing, echolalia and echopraxia.^3,4^ According to this latter definition, three or more out of twelve established clinical features (among all characteristics already cited) are necessary to establish a diagnosis of catatonia.^3,4^ However, some specialists criticize this new DSM-5 criteria as it lists inaccurate motor and speech abnormalities which overlap with various neurological and psychiatric disorders,^1^ possibly causing confusion in determining catatonia as an entity in itself.

Catatonia etiology can be structural or functional diseases of the central nervous system (CNS) as well as systemic disease causing neurological symptoms. Carroll et al. (1994) studied a group of catatonic patients and found that, of those cases with an apparent psychiatric etiology, around a third had a neurological condition as the presumed cause.^5^ In the cited casuistic, of ten patients with seizure disorders, nine had encephalitis or another CNS infection.^5^ Huang et al. (1999) found that 41% of Chinese patients had general medical conditions underlying catatonic features.^6^

In a 2015 review of catatonia, the most frequent underlying causes found, besides schizophrenia and mood disorders, were anti-N-Methyl-D-Aspartate Receptor (NMDAR) encephalitis, systemic lupus erythematosus or antiphospholipid syndrome, infectious encephalitis, autism and complications secondary to liver disease or kidney transplantation.^1^

In addition, the association between catatonia and seizures in patients with neurological disorders is well known.^1^ In general, eight to 15% of all cases diagnosed as catatonia have an associated epileptic condition.^5^ When a patient has an ACS it is necessary to search for epileptic manifestations by performing EEG and determining whether there is concomitant electrical brain activity (EBA). If EEG shows EBA in the course of clinical features, this characterizes catatonia as an ictal manifestation.^7^

Moreover, 'ictal catatonia' may be part of NCSE.^8^ However, literature lacks precise data on the prevalence of catatonia as part of NCSE, since it is difficult to perform EEG when clinical sign are present, which unfortunately was also the case in our patient. Consequently, only case series or case reports in small casuistics are available. Lim et al. (1986) reported three cases of ictal catatonia with an appropriately conducted EEG analysis that fulfilled NCSE criteria.^8^

Although an EEG without EBA in the presence of catatonia excludes 'ictal catatonia', other seizures can also occur, rarely before or more commonly after catatonia manifestation.^7,9^ Primavera et al. (1994) published a case series in which 13.8% had seizures after ACS onset, and the majority of patients had viral encephalitis.^9^

On the other hand, catatonia can be a clinical feature of cerebral ischemic infarction generally.^1^ The presentation due to a CVST for instance is rare. Moreover, catatonia has also been associated with subarachnoid hemorrhage and subdural hematoma.^10,11^ However, its incidence in cerebrovascular cases overall is not precisely defined.

CVST diagnosis is a 'chameleon' as it can mimic many different conditions in neurology. The most frequent symptom is headache (around 80%).^11^ Convulsive seizures^7^ and cranial nerve dysfunction^11^ can also be frequently seen. Although rare in presentation, psychiatric disorders are among possible related symptoms.^11^ To our knowledge, there is only one case of CVST occurring with catatonia as the initial presentation described in the literature to date.^10^ A case report of neuropsychiatric symptoms associated with CVST was also published in a web-journal in 2013.^11^

The complete physiopathology of catatonic syndrome and its correlation with cerebral structures are not fully understood. Some studies suggest hypometabolism in the basal ganglia,^12^ while others show hypoperfusion in frontal, temporal and parietal lobes.^13,14^ Functional imaging studies for instance have shown that catatonia is associated with altered activity in orbito-frontal, prefrontal, parietal, and motor cortical regions, where these cortical structures may play a role in the pathophysiology of catatonia.^15^ This is also reinforced by observations that GABA-A binding is reduced in cortical areas.^7,15^ There may also be dopaminergic involvement related to the diencephalon, brainstem and limbic regions, such as the cingulate gyrus.^16^

In our case, catatonia may have been the clinical manifestation of a cerebral infarction in the left -posterior medial temporal area due to a CVST or a possible epileptic manifestation that occurred secondary to this brain injury, or perhaps the result of both. A study limitation was that no EEG was performed during the clinical manifestation of catatonia to establish whether there was in fact epileptic activity at the time. Therefore, it is not possible to confirm this epileptic hypothesis, although the clinical course suggested 'ictal catatonia' given that the patient's behavioral symptoms improved and did not recur after antiepileptic medication infusion at the Emergency Department.

## CONCLUSION

If catatonia presents acutely, complete history with a thorough semiology analysis of patient symptoms and further complementary investigation are always necessary. The primary goal is to search for an epileptic event with nonconvulsive manifestation. We must change the misconception that catatonia is solely a psychiatric illness. In fact, it is a condition with many possible etiologies whose prognosis depends on correct differential diagnosis that allows the physician to provide early treatment of its underlying cause.
